# The effect of ECD program on the caregiver’s parenting knowledge, attitudes, and practices: based on a cluster-randomized controlled trial in economically vulnerable areas of China

**DOI:** 10.1186/s12889-022-14268-5

**Published:** 2022-10-24

**Authors:** Ying Li, Shanshan Li, Lei Tang, Yu Bai

**Affiliations:** 1 School of Public Administration, Zhongnan University of Economics and Law, 430073 Wuhan, Hubei Province China; 2 Innovation and Talent Base for Income Distribution and Public Finance, Zhongnan University of economics and law, 430073 Wuhan, China; 3 Center for Experimental Economics in Education, Shaanxi Normal University, 710119 Xi’an, Shaanxi Province China; 4 School of Economics & China Institute for Vitalizing Border Areas and Enriching the People, Minzu University of China, 100081 Beijing, China; 5 Peking University, 100871 Beijing, China

**Keywords:** Early childhood development, Parenting knowledge, Parenting attitudes, Parenting practices, Economically vulnerable areas, Randomized controlled trial

## Abstract

**Background:**

The first three years of life are the critical and sensitive periods for the formation of individual abilities. However, existing data indicates that early childhood development (ECD) in economically vulnerable areas of China is lagging, which is closely related to the lack of parenting knowledge and poor parenting practices.

**Methods:**

We conducted a non-masked cluster-randomized controlled trial in a former nationally designated poverty county of China. All 6–36-month-old children and their caregivers living in 18 communities/clusters (10 towns and 8 districts of the county seat) were enrolled in a 9-month parenting training program. In the treatment-group communities, ECD centers were installed where community workers provided parenting training sessions. If caregivers were unable to visit the center, home-based parenting training was offered. No intervention was provided to the control group. Furthermore, we assigned half of the treatment group to receive monthly developmental feedback in addition to the parenting training. Based on the baseline and follow-up data, we investigated the treatment effects on parenting knowledge, attitudes, and practices through *Intention-to-Treat* (ITT) and *Treatment-on-the-Treated* (TOT) analyses.

**Results:**

We found no effects on the parenting knowledge and attitudes of the caregivers but significant effects on the parenting practices. The effects were heterogeneous among families with different characteristics. Specifically, on average, the program had the largest effect on internally oriented caregivers, mothers with higher education, and mothers who are primary caregivers. We want to emphasize that, although the ITT effect on parenting practices (the average treatment effect) were stronger for mothers with higher education, the TOT effect on parenting practices (the local average treatment effect, LATE) were stronger for mothers with less education. That is, even though on average the program helped mothers with higher education, but among complier families, the program benefited mothers with less education.

**Conclusion:**

The findings indicate that, at least in the short run, the program can directly change caregivers’ parenting practices without changing their knowledge and attitudes. Future studies are needed to investigate whether parenting knowledge and attitudes can change in the long run.

**Supplementary Information:**

The online version contains supplementary material available at 10.1186/s12889-022-14268-5.

## Introduction

The human brain and nervous system undergo rapid changes during early childhood. Early childhood development, such as, motor, language, cognition, social emotion, and other fields (Aboud and Yousafzai 2015) can predict children’s future academic performance, human capital accumulation, and their adulthood income levels (Engle et al. 2007; Black et al. 2008; Currie and Almond 2011). However, existing studies show that approximately 249 million children under the age of five in low- and middle-income countries (hereinafter referred to as “LMICs”) are at risk of poor development, of which 17.43 million (roughly 8%) are in China, ranking second in the world (Lu et al. 2016). The ECD problem is particularly severe in economically vulnerable areas in China. According to an empirical study, half of the children living in rural China are at risk of cognitive developmental delay, and 52% are at risk of language developmental delay. These risks will increase over time if no measures are taken (Yue et al. 2019).

Children can make significant progress in motor, language, cognitive, and socio-emotional development if properly cared for (Grover 2005). Caregivers are the primary providers of stimulating and supportive experiences related to ECD (Britto et al. 2002; Richter 2004; Bradley and Corwyn 2005). However, in LMICs, children are exposed to a variety of psychosocial risk factors for not receiving appropriate care, including insufficient stimulation, ineffective parenting practices, unresponsive care, and the inability of parents/caregivers to understand infant behaviors (Wallander et al. 2014; Yue et al. 2017; Li et al. 2019), all of which can have a detrimental impact on a child’s development.

The above risk factors can be classified as lack of parenting knowledge, attitude, and practices. Parenting knowledge is defined as an understanding of child development norms, milestones, developmental processes, and familiarity with parenting skills (Benasich and Brooks-Gunn 1996), and it is a predictor of child development (Huang et al. 2005). Existing studies have not only found a direct link between parenting knowledge and children’s outcomes, including reduced behavioral problems and improved cognitive and motor performance (Benasich and Brooks-Gunn 1996; Dichtelmiller et al. 1992; Rowe et al. 2015), but have also indicated that parenting knowledge and beliefs have a continuous and stable influence on children’s outcomes (Zigler 1992). The mechanisms are that parenting knowledge influences a child’s outcomes by shaping the family environment and improving the quality of parenting practices (Huang et al. 2005; Sajedi et al. 2016). Knowledgeable mothers are more likely to provide their children with books and learning materials suitable for their children’s interests and age, and knowledgeable mothers are more likely to read, talk, and tell stories more to their children (Grusec 2011; Gardner-Neblett et al. 2012). Moreover, knowledgeable mothers may provide a warm and positive environment that promotes children’s social-emotional development (Smith 2002).

Parenting attitudes are the product of parenting knowledge, parenting values, and goals (or expectations) for their children’s development. These values ​​and goals are, in turn, influenced by cultural, social, and parental experiences and their overall values and goals (Rogoff 2003; Okagaki and Bingham 2005; Iruka et al. 2015). To a certain extent, the formation of attitudes is determined by parental self-efficacy, that is, the ability of parents to perceive their influence on the child’s development. Parental self-efficacy affects parenting abilities, parenting practices, and children’s abilities (Jones and Prinz 2005).

Parenting practices refer to parent-child activities, such as reading, singing, playing with children, and cultivating a child’s sense of discipline. Previous studies have shown that parenting practices play an important role in individual development (Evens et al. 2000; Black et al. 2017; Yue et al. 2017). For example, Bai et al. (2019) found that the better the parent-child interactions and discipline behaviors, the lower the risk of delayed child development. In the short-term, positive parenting practices can stimulate and promote children’s cognitive and language development as well as stimulate and maintain children’s enthusiasm and interest in learning. In the long term, it has a positive impact on children’s early literacy, academic performance, and future life happiness (Darling and Steinberg 1993; Mulvaney et al. 2006; Keels 2010; Page et al. 2010).

Due to the positive role of parenting knowledge, attitudes, and quality practices in child development (NASEM, 2016), the *Lancet* ECD series has embraced nurturing care as the basis for successful ECD strategies (Britto et al. 2017). Most ECD programs in LMICs focus on psychosocial stimulus interventions that are typically directed at encouraging parents to provide children with opportunities to explore their surroundings, solve problems, and interact with others (Jeong et al. 2018). These projects are effective in improving child development (Yousafzai and Aboud 2014; Yousafzai et al., 2014; Aboud and Yousafzai 2015; Britto et al. 2015, 2017; Rao et al. 2017). However, it should be noted that the key to the success of these programs is their ability to change the knowledge, beliefs, attitudes, and practices of caregivers.

Some studies using randomized controlled trial methods have found that ECD interventions not only have a positive impact on parents’ knowledge of child development but also have an impact on caregivers’ practices in caring and feeding their children (Alkon et al. 2014; Yousafzai et al. 2015). Significant benefits for mothers’ parenting practices have also been found in some ECD interventions aimed at improving parent-child interactions by promoting mothers’ sensitivity and responsiveness to infants (Cooper et al. 2002, 2009). Previous research has also shown that providing caregivers with opportunities to learn how to observe and respond to their children through games and communication interactions can improve parenting knowledge, the quality of the family environment, parenting involvement in child development, and parent-child interactions (Yousafzai et al. 2015).

Some studies on disadvantaged children and their families in developing countries have found significant impacts on mothers’ knowledge of child development (Rahman et al. 2008; Powell et al. 2004; Aboud 2007; Jin et al. 2007). An analysis of 34 home visits for high-risk infants revealed that home-based parenting interventions continuously improved the family environment and parenting skills (Kendrick et al. 2000). Similarly, a review of six large-scale home visit programs in the United States found that home-based parenting interventions have a positive impact on parenting attitudes and practices, especially for those who need them the most (Gomby et al. 1999). Another study of preterm infants found that home-based interventions can promote more sensitive and responsive parenting skills, thereby improving parent-child interactions (Goyal et al. 2013). A systematic review also found that ECD interventions in LMICs targeting children under two years of age have positive impacts on parenting outcomes. Specifically, the interventions had medium-to-large positive effects on the home care environment, parenting knowledge, and mother-child interactions (Jeong et al. 2018).

While previous studies have investigated the effect of ECD interventions on parenting knowledge, attitudes, and practices, to our knowledge, there has been no comprehensive evaluation of the effect of such ECD intervention programs on the improvement of parenting skills or capacities in China, which is the key mechanism for the success of ECD programs (Jeong et al. 2018). Moreover, parents from various cultural backgrounds have different expectations of their children’s socialization, parenting attitudes, and practices (NASEM, 2016), which may result in different effects on children’s development. In addition, few studies have examined such effects of government-led, multi-delivery models, all-inclusive, and large-scale ECD programs.

In recent years, the Chinese government has released several policy documents to emphasize the importance of early development of children under the age of three. However, the implementation of this policy remains to be explored. This study used a non-masked cluster-randomized controlled trial to assess the causal effects of a government-led ECD intervention project implemented in economically vulnerable areas in China on caregivers’ parenting knowledge, attitudes, and practices. The rationale of assigning treatments on cluster levels is to avoid contamination. We believe that the results of this study will be of great significance for not only the Chinese government but also other countries in a similar situation to formulate comprehensive intervention policies to promote ECD in rural areas.

## Data and methods

### Trial design

This cluster-randomized controlled trial was conducted in a former, nationally designated poverty county in Shaanxi Province, China. To minimize the risk of contamination across the treatment and control groups, we randomized the treatment (i.e., delivery of weekly parenting training) at the community level among 18 communities/clusters (10 towns and 8 districts of the county seat). The treatment group was randomly divided into two treatment arms: “feedback” and “no feedback.” These two treatment arms were enrolled for a weekly parenting training program, but the feedback arm received monthly feedback on the child’s developmental progress on the top of the parenting training program. The control group did not receive any treatment. The intervention was implemented for nine months, from July 2018 to March 2019. A baseline survey was conducted in June 2018, followed by a follow-up survey in April 2019.

## Randomization and masking

In January 2018, the research team generated a random allocation sequence using the STATA program for the random assignment of the communities into control and treatment groups. Furthermore, half of the treatment group were randomly assigned to the two (i.e., “feedback” and “no feedback”) treatment arms at the individual level and stratified by the children’s levels of development (i.e., the baseline scores of *Ages and Stages Questionnaires, Third Edition* (ASQ-3)). The reason of stratification is to ensure balance of the treatment arms and to increase efficiency. In June 2018, the research team enrolled participants and assigned them to interventions at the community level. Although complete masking was not possible in this study, the caregivers and parenting trainers were unaware that they were involved in the experiment. Furthermore, the survey teams were blinded to the group assignments.

## Sample size determination

The sample size was determined using a power calculation with a detectable effect size on the main outcome variable of interest at 80% power, given a two-sided significance level of 0.05. Allowing for an attrition rate of 10%, based on evidence from earlier field experiments in rural China, we assumed an adjusted intraclass correlation coefficient (ICC) of 0.01 and that baseline scores account for 50% of the variation in scores at follow-up. Based on these parameters, we calculated that nine clusters of 35 children per treatment arm would allow us to detect an effect size of 0.24 SD at 80% power, given a two-sided significance level of 0.05.

## Participants

**Fig. 1:**
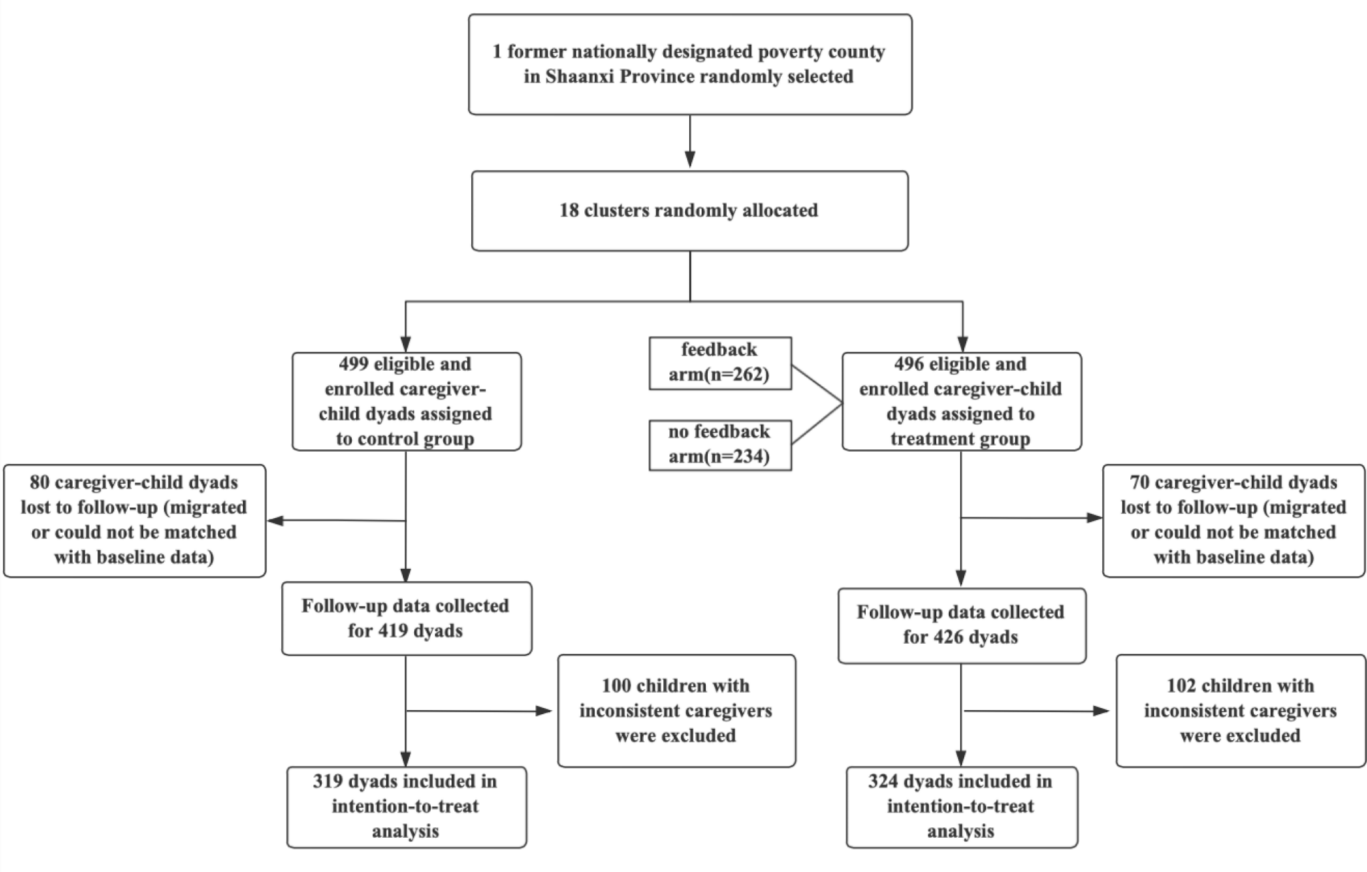
Trial profile

We obtained the birth registration of the study county from the county-level office of the National Health Commission (NFC) and recruited all children aged 6–36 months and their primary caregivers. Birth registration was confirmed by local councilors and the research team. On average, 14 children were enrolled in each cluster or community. As of June 2018, the baseline sample included 995 child-caregiver dyads from 18 communities. Due to migration, illnesses, or short-term leave of residence (such as visiting relatives in other places during the survey period), the sample attrition rate was approximately 15%. The resulting follow-up sample included 845 child-caregiver dyads as of April 2019. Furthermore, we excluded samples with inconsistent types of caregivers in the baseline and follow-up surveys, and the final sample for analysis included 643 child-caregiver dyads. The trial profile of this study was as follows (see Fig. 1):

All study protocols were approved by the institutional review boards (IRBs). All subjects provided written informed consent to participate in the experiment and data collection before the commencement of the project. There was no known harm or risks to the participants. The development of research questions and outcome measures was not influenced by participants’ priorities, experiences, and preferences. Research reports were available to participants upon request.

### Intervention and implementation

All children and their caregivers in the treatment group were enrolled in a government-led ECD intervention project. The intervention focused on child psychosocial stimulation (hereafter referred as “weekly parenting training”). These services were delivered by locally employed and project-trained parenting trainers who had educational attainments at the level of the mothers that they provided the services. The local government was in charge of recruiting and hiring parenting trainers as well as the daily management and supervision of the delivery of this program. The research team had limited involvement in program delivery and focused only on the curriculum development and training of parenting trainers.

Studies have shown that center-based interventions can improve early childhood development, including social-emotional skills and the primary caregiver’s well-being (Engle et al. 2011; Singla et al. 2015). However, the most vulnerable children and marginalized populations may not benefit from a center-based model because they typically live in remote areas. It has been found that ECD projects based on home visits can support the most disadvantaged children more effectively. Studies conducted in economically vulnerable areas of China have shown that both center- and home-based service models are effective (Li 2020; Zhong et al. 2020). To optimize the cost-effectiveness, dosage, and coverage of the program, the services were delivered using a mixed intervention model:

#### Model 1: ECD center + home visit

An ECD center is installed if a village has at least ten infants and toddlers ages 6–36 months within one kilometer of the selected program site in the village center. These centers are open six days a week (from Monday to Saturday), six hours a day. Each center has separate areas or rooms for one-to-one training courses, reading activities, and a spacious play area for parent-child activities. All services, books, and toys were provided free of charge to registered families in the communities.

The weekly course schedule was as follows. First, a weekly parenting training course provided one-to-one parenting training to the caregivers. A caregiver-child dyad is required to attend the course together. The course focuses on demonstrating and teaching the caregiver about the age-appropriate child psychosocial stimulation activities and encouraging caregiver-child interactions. The course material is a designated curriculum for the National Health Commission’s Cadre Training Center project, which aims to promote cognitive, language, motor, and social-emotional development. Second, group playing and reading activities were organized weekly by parenting trainers. Finally, parenting trainers encourage caregivers to borrow toys and books after each weekly session and to practice activities at home.

For a few families that cannot come to the center on a regular basis due to distance, caregivers who are older adults, caring for multiple children, or having physical health problems, parenting trainers will bring the relevant toys and books with them and deliver the one-to-one parenting training course during their weekly home visits.

#### Model 2: home visits + family group activity

Parenting service points are installed in villages where the number of nearby children is inadequate to establish a center. One parenting trainer is employed for each service point to provide weekly one-to-one parenting training services to eligible families during home visits. The parenting trainer also organizes group activities for two hours per week if there are two or more families living in the service areas.

Among families in the treatment group, 14.34% received Model 2 (home-based) treatment and 85.66% received Model 1 (center-based) treatment. The project team developed a supervision system to ensure the quality-of-service delivery. This supervision system includes an IT management system and a local staff team that performs daily management of the project. The IT system records the registration information of families, family class attendance, video records of one-to-one classes, group activities, and other information. The project staff is responsible for conducting regular face-to-face supervision to parenting trainers and follow-up phone calls families served by the program.

For the feedback arm, parents and main caregivers received monthly feedback on their child’s developmental progress and the child’s relative ranking among peers via phone messages on top of parenting training services.

## Outcome measures

The primary outcomes of this study were measures of parenting knowledge, attitudes, and practices. These measures were included in a comprehensive household questionnaire and administered during one-to-one interviews with primary caregivers at their home. Interviewers used a Computer-Assisted Personal Interviews (CAPI) system to record caregivers’ answers. In addition, the instruments, such as ASQ and CREDI, were used to measure the child development status and were reported by caregivers.

### Parenting knowledge

The *Knowledge of Infant Development Inventory-P* (KIDI-P) was used to measure the caregivers’ parenting knowledge levels. This questionnaire is a simplified version of the KIDI. Both the KIDI and KIDI-P have been widely used in research on parenting knowledge and child development (Huang et al. 2005; Zolotor et al. 2008; Al-Maadadi and Ikhlef 2015). During the interviews, the main caregivers were asked to indicate whether they agreed or disagreed with 58 ECD-related statements. Raw item scores were used to calculate the ratio of correct responses. The ratio varied from zero to one. Higher ratios represented better parenting knowledge of the ECD process and milestones.

#### Parenting attitudes

The *Parental Locus of Control scale* (PLOC) developed by Campis et al. (1986) was used to measure caregivers’ parenting attitudes. The PLOC measures the degree to which parents believe that their children’s future skill development is either a matter of chance or fate (external orientation) or the result of parenting investment and practices (internal orientation). Internal orientation is regarded as a positive trait because it represents the belief that personal efforts are primarily responsible for the results of life. The scale has four dimensions: parental efficacy, parental responsibility, children’s control of parents’ lives, and parental control of children’s behavior. Each dimension had six items with the highest factor loadings. The main caregivers were asked to rate how much they agreed with the statements of each item on a five-point Likert scale. A higher score of “parental efficacy” indicates a lower sense of efficacy, a higher score of “parental responsibility” indicates a lower sense of responsibility for their children’s behavior, a higher score of “child control of parent’s life” indicates a stronger belief that children are in control of parents’ lives, and a higher score of “parental control of child’s behavior” indicates a weaker belief that parents can control their children’s behavior. Therefore, a higher (lower) score on each dimension indicates a more external (internal) orientation.

#### Parenting practices

We have two measures of parenting practices: caregiver-child interactions and child behavior discipline styles. Caregiver-child interactions were measured using six questions from the *Family Care Indicators* (FCI) adapted from UNICEF’s Home Observations for Measurement of Environment (Bradley, 1977). The six questions were: (1) Did you read a book or read a picture book to your baby in the past three days? (2) Did you tell your baby a story in the past three days? (3) Did you sing nursery rhymes to your baby in the past three days? (4) Did you take your baby to play games outdoors in the past three days? (5) Have you played games with your baby with toys in the past three days? (6) Did you spend time with your baby naming things, counting, or drawing pictures in the past three days?

Disciplining child behavior is an essential part of raising children in all cultures. It teaches children self-control and acceptable behavior (UNICEF, 2010). According to the Multiple Indicator Cluster Surveys (MICS) and the Demographic and Health Surveys (DHS) conducted in 2005 and 2006, non-violent discipline behaviors are more common than violent ones in most countries and are closely related to authoritative parenting practices, such as depriving rights or explaining why something is wrong. Children raised by authoritative parents are more goal oriented and independent (Baumrind 1978, 1991). In this study, three questions were used to measure discipline styles: 1. When you discipline your baby, do you take away toys or other things your baby wants? 2. When you discipline your baby, do you limit time to stop the baby’s behavior? 3. When you discipline your baby, do you explain why a behavior is unacceptable? The main caregivers were asked to indicate whether the stated situation happened “often,” “sometimes,” “rarely,” or “never”. A higher score for each question indicated a lower frequency of stated discipline styles.

#### ECD outcomes

The ECD level was assessed using the *Ages & Stages Questionnaires, Third Edition* (ASQ-3) (Squires et al. 2009), *Ages & Stages Questionnaires: Social-emotional* (ASQ: SE) (Squires et al. 2002), and the long form of the *Caregiver Reported Early Childhood Development Instruments* (CREDI) (McCoy et al. 2017). We aggregated the scores of the different domains of these surveys to generate three developmental measures: cognitive, noncognitive, and physical development. According to Doyle et al. (2017), cognitive and language scores are indicators of latent variables of cognitive development, social-emotional and behavioral scores are indicators of latent variables of non-cognitive development, and gross motor and fine motor scores are indicators of latent variables of physical development. The factor analysis method (Heckman et al. 2010) was used to determine the potential factors of the three developmental measures. We first performed standardization processing based on the original scores of each domain in each ECD scale, we then extracted the estimated mean and factor loads through factor analysis, and finally, we used the Bartlett scoring method to predict each child’s factor score.

Improving ECD outcomes is the aim of our intervention but is not the emphasis of the current study. ECD outcomes are included in the analysis as baseline control variables. The research team is still working on the impact of intervention on ECD outcomes. To facilitate readers to understand the baseline ECD status, please see the Appendix Fig. 1 and Appendix Table1 and the associated notes.

**Table 1:** Baseline characteristics of the participants in the treatment and control group

	Control	Treatment	p-value
***Individual characteristics***			
Gender	0.484	0.489	0.888
	(0.025)	(0.018)	
Months	20.594	20.139	0.455
	(0.513)	(0.370)	
Premature	0.041	0.044	0.878
	(0.008)	(0.012)	
Minority	0.085	0.008	0.046
	(0.038)	(0.004)	
The child was firstborn	0.745	0.780	0.276
	(0.026)	(0.021)	
Low birth weight ( < = 2.499 kg)	0.043	0.045	0.863
	(0.007)	(0.010)	
The first factor of cognitive development	0.026	0.004	0.824
	(0.066)	(0.073)	
The first factor of non-cognitive development	0.111	-0.082	0.073
	(0.081)	(0.084)	
The first factor of physical development	0.072	-0.046	0.269
	(0.070)	(0.081)	
***Family characteristics***			
The primary caregiver is a mother	0.718	0.667	0.172
	(0.022)	(0.031)	
Mother’s education level (> 9 years of formal education)	0.450	0.443	0.933
	(0.080)	(0.063)	
Age of the mother (years)	29.232	29.267	0.957
	(0.511)	(0.375)	
Hukou of parents	1.397	1.354	0.784
	(0.126)	(0.114)	
Whether is *Dibao (low income family)*	0.112	0.124	0.738
	(0.029)	(0.021)	
Distance from the home to township seat	1.516	1.561	0.859
	(0.156)	(0.205)	
N	483	481	

Notes for variable Minority: China is a unified multi-ethnic country. Due to the large population of Han ethnicity (over 90% of the country’s total population), it is customary to refer to the other 55 ethnic groups as ethnic minorities. In terms of differences among the ethnicities, Han ethnicity speak Chinese, and some ethnic minorities have their own languages and scripts. In addition, there are differences between Han and some ethnic minorities in life customs, values, folk customs, and etiquette. In terms of geographical area of residence, most ethnic minorities are distributed in frontier provinces, and some of them migrate to live in Han-dominated regions. In the sampled area of this study, there is a very small number of Hui ethnicity. These few Hui people have been migrated to the sampled area and integrated into local cultures for a long time, thus, there is no significant differences in terms of language, values, and customs between these minorities and the Han ethnicity. The Chinese government does not discriminate in preferential policies neither. Therefore, in terms of the intervention, the two groups received the identical treatments.

### Statistical analysis

We used Stata 15.1 for statistical analysis. First, we summarized the descriptive statistics of the treatment and control group data at baseline and follow-up, respectively. A balance test was performed between the treatment and control groups at baseline and follow-up.

Second, we estimated *Intention-to-Treat* (ITT) effects of the intervention on outcome variables (i.e., caregivers’ parenting knowledge, attitudes, and practices). The model is expressed as follows:

$${Y}_{ijt}=\alpha +\delta {Y}_{0jt}+\beta {W}_{j}+{X}_{ij}+{E}_{i}+{\epsilon }_{ijt}$$

where ${Y}_{0jt}$ and ${Y}_{ijt}$ represent the level of the outcome variables of individual *i* in family *j* at baseline and follow-up, respectively. ${W}_{j}$ is a dummy variable for whether family *j* was assigned to the treatment group, with ${W}_{j}=1$ if the family was assigned to the treatment group and ${W}_{j}=0$ otherwise. ${X}_{ij}$ represents individual and family characteristics. ${E}_{i}$ is the enumerator fixed-effect. ${\epsilon }_{ijt}$ is the random error term. $\beta$ is the treatment effect.

Third, due to incomplete compliance, we estimate *Treatment-on-the-Treated* (TOT) effects to evaluate treatment effects on compliers (i.e., those who were not only assigned to the treatment but also participated in at least one intervention activity). The TOT effect was estimated using the instrumental variable method. The first-stage regression model is as follows:

$${T}_{i}={\alpha }_{0}+{\alpha }_{1}{W}_{i}+{\alpha }_{i}{X}_{i}+e$$

Among them, ${T}_{i}$ refers to whether a family received the treatment (i.e., participating in at least one intervention activity), and ${W}_{i}$ is an instrumental variable that represents whether a family is assigned to the treatment group.

We then predict the probability of receiving the treatment as follows:

$$\widehat{{T}_{i}}=\widehat{{\alpha }_{0}}+\widehat{{\alpha }_{1}}\widehat{{W}_{i}}+\widehat{{\alpha }_{i}}\widehat{{X}_{i}}$$

The second stage regresses the outcome variable ${Y}_{i}$ on the predicted probability of receiving treatment $\widehat{{T}_{i}}$:

$${Y}_{i}={\beta }_{0}+{\beta }_{1}\widehat{{T}_{i}}+{\beta }_{i}{X}_{i}+{\epsilon }_{i}$$

Among them, ${\beta }_{1}$is the TOT effect.

Fourth, we investigated heterogeneous ITT and TOT effects for four subgroups: internal vs. external locus of control caregivers, high- vs. low-education mothers (i.e., junior high school or below vs. above junior high school), mothers as main caregivers vs. non-mothers as caregivers, and center-based vs. home-based delivery models.

Fifth, we used the difference-in-differences (DID) method as a robustness check to evaluate changes in parenting knowledge, attitudes, and behaviors during the baseline and follow-up surveys.

In all regressions, we controlled for baseline parenting knowledge, attitudes and practices, child characteristics, and family characteristics. The scores for parenting knowledge and attitudes were internally standardized. All standard errors were clustered at the village level. We also adjusted the multiple hypotheses testing problem using the step-down procedure of Romano and Wolf (2005) to control for the family wise error rate (FWER).

## Results

### Balance test results

At baseline, 995 children were sampled, with 499 children in the control group and 496 in the treatment group. A total of 150 samples were attritted during the follow-up survey, with 70 from the treatment group and 80 from the control group. Furthermore, we excluded 202 participants whose caregivers’ types were inconsistent between the baseline and follow-up surveys, with 102 from the treatment group and 100 from the control group. Table 1 shows the basic individual and family characteristics of the treatment and control groups. At baseline, 668 children were primarily cared for by their mothers (69.29%), with 321 (66.7%) in the treatment group and 347 (71.8%) in the control group. In addition, 296 children were primarily cared for by others (30.71%), most of whom were older and less educated grandparents (please see Appendix Table 2for information on the differences between mother and non-mother caregivers). Overall, there was no systematic imbalance in most baseline covariates between the treatment and control groups. Considering the few significant results, the individual characteristics at baseline are incorporated into the model to address the possible estimation bias.

**Table 2:** Baseline parenting knowledge, attitudes, and practices in the treatment and control group

	Control	Treatment	p-value
***Knowledge***			
KIDI total score	0.518	0.504	0.031
	(0.004)	(0.005)	
***Attitude***			
**PLOC overall**	65.209	65.630	0.139
	(0.245)	(0.201)	
PLOC parental efficacy	15.505	15.814	0.081
	(0.133)	(0.117)	
PLOC parental responsibility	16.111	16.280	0.439
	(0.155)	(0.145)	
PLOC child control of parent’s life	15.215	15.236	0.879
	(0.109)	(0.093)	
PLOC parental control of child’s behavior	18.378	18.312	0.754
	(0.123)	(0.166)	
***Practices***			
***Parent-child interactions***			
**Total parent-child interactions**	3.476 (0.167)	3.348 (0.162)	0.555
Reading books	0.414	0.457	0.436
	(0.045)	(0.035)	
Telling stories	0.365	0.346	0.676
	(0.039)	(0.030)	
Singing songs	0.646	0.566	0.139
	(0.044)	(0.035)	
Taking child outside the home for play	0.842	0.809	0.317
	(0.022)	(0.024)	
Playing with the child with toys	0.783	0.717	0.065
	(0.029)	(0.026)	
Naming things, counting, drawing	0.425	0.453	0.502
	(0.025)	(0.034)	
***Disciplining practices***			
**Total discipline practices**	7.068 (0.123)	7.069 (0.111)	0.992
Taking away children’s things	2.688	2.730	0.418
	(0.036)	(0.039)	
Limiting time	2.660	2.679	0.874
	(0.090)	(0.073)	
Explanation	1.719	1.660	0.388
	(0.052)	(0.044)	
N	483	481	

Table 2 reports the baseline levels of parenting knowledge, attitudes, and practices of the caregivers in the treatment and control groups, respectively. Most variables between the treatment and control groups are not significantly different.

Furthermore, due to sample loss and data exclusion, a balance test is needed to assess whether the treatment and control groups are still in balance after sample loss and exclusion. Appendix Tables 3 and 4 show the results of the balance tests for baseline characteristics, parenting knowledge, attitudes, and practices. The results in Appendix Table 3 show that most of the characteristics were not significantly different between the treatment and control groups. Despite this, baseline child development levels were controlled in the analysis to adjust for potential estimation bias. The results in Appendix Table 4 show that most variables of parenting knowledge, attitudes, and practices were insignificantly different between the treatment and control groups, except for a few variables. As a result, baseline parenting knowledge, attitudes, and practices were included in the analysis to account for possible estimation biases.

**Table 3:** ITT analysis of treatment on parenting knowledge, attitudes, and practices

	Treatment effect			
	Coefficients		SE	Romano-Wolf P-value
**Panel A. Parenting Knowledge(N = 563)**				
KIDI total score	0.031		0.067	0.951
**Panel B. Parenting Attitude (N = 563)**				
PLOC overall	0.012		0.084	1.000
PLOC parental efficacy	0.113		0.079	0.406
PLOC parental responsibility	0.015		0.082	1.000
PLOC child control of parent’s life	0.004		0.062	1.000
PLOC parental control of child’s behavior	-0.088		0.103	0.762
**Panel C. Parenting Practices(N = 564)**				
***Parent-child interactions***				
Reading books	0.126		0.050	0.030
Telling stories	0.136		0.046	0.010
Singing songs	0.064		0.040	0.319
Taking child outside the home for play	-0.037		0.036	0.762
Playing with the child with toys	0.049		0.033	0.406
Naming things, counting, drawing	0.146		0.038	0.010
***Disciplining practices***				
Taking away children’s things	-0.060		0.060	0.762
Limiting time	-0.101		0.089	0.703
Explanation	-0.051		0.066	0.772

**Notes**: In all regressions, we controlled for baseline parental knowledge, attitude and practices, child characteristics, and family characteristics. The scores for parental knowledge and attitudes were internally standardized. All standard errors were clustered at the village level. We also adjusted the multiple hypotheses testing problem using the step-down procedure of Romano and Wolf (2005) to control for the family wise error rate (FWER). The significance levels are as follows: *p < 0.1, **p < 0.05, and ***p < 0.01

**Table 4:** ITT analysis of treatment on parenting knowledge, attitudes, and practices by the feedback type

	Feedback					No Feedback				
	Coefficients	SE		Romano-Wolf P-value		Coefficients		SE		Romano-Wolf P-value
**Panel A. Parenting Knowledge (N = 563)**										
KIDI total score	0.034	0.071		0.980		0.027		0.086		1.000
**Panel B. Parenting Attitude (N = 563)**										
PLOC overall	0.003	0.097		1.000		0.024		0.096		1.000
PLOC parental efficacy	0.165	0.087		0.181		0.050		0.081		1.000
PLOC parental responsibility	0.013	0.107		1.000		0.018		0.109		1.000
PLOC child control of parent’s life	-0.014	0.075		1.000		0.027		0.063		1.000
PLOC parental control of child’s behavior	-0.114	0.116		0.842		-0.055		0.106		1.000
**Panel C. Parenting Practice(N = 564)**										
***Parent-child interactions***										
Reading books	0.135	0.057		0.040		0.115		0.053		0.099
Telling stories	0.131	0.052		0.020		0.142		0.050		0.030
Singing songs	0.042	0.045		0.842		0.092		0.049		0.198
Taking child outside the home for play	-0.050	0.038		0.525		-0.020		0.045		1.000
Playing with the child with toys	0.088	0.039		0.069		-0.001		0.038		1.000
Naming things, counting, drawing	0.156	0.052		0.010		0.133		0.044		0.020
***Disciplining practices***										
Taking away children’s things	0.016	0.060		1.000		-0.156		0.082		0.188
Limiting time	-0.061	0.090		0.931		-0.151		0.110		0.475
Explanation	-0.037	0.068		0.980		-0.070		0.082		0.941

**Notes:** In all regressions, we controlled for baseline parental knowledge, attitude and practices, child characteristics, and family characteristics. The scores for parental knowledge and attitudes were internally standardized. All standard errors were clustered at the village level. We also adjusted the multiple hypotheses testing problem using the step-down procedure of Romano and Wolf (2005) to control for the family wise error rate (FWER). The significance levels are as follows: *p < 0.1, **p < 0.05, and ***p < 0.01

## Exposure to treatment

Appendix Table 5 provides an overview of the completion of intervention activities. On average, caregiver-child dyads of the treatment group completed 10.59 (9.13 SDs) one-to-one parenting training sessions. They visited the ECD centers 2.93 (31.32 SDs) times and spent 29.91 (48.53 SDs) hours at the ECD center. Due to noncompliance in the control group, families of the control group completed 1.03 (3.38 SDs) of one-to-one sessions on average, they visited the ECD centers 3.14 (10.64 SDs) times, and they spent 3.39 (11.16 SDs) hours at the ECD centers.

**Table 5:** TOT analysis of treatment on parenting knowledge, attitudes, and practices

	Whether attended one-to-one session			
	Coefficients		SE	Romano-Wolf P-value
**Panel A. Parenting Knowledge(N = 548)**				
KIDI total score	0.035		0.094	0.980
**Panel B. Parenting Attitude (N = 563)**				
PLOC overall	0.016		0.116	0.980
PLOC parental efficacy	0.163		0.107	0.327
PLOC parental responsibility	0.013		0.117	0.832
PLOC child control of parent’s life	0.023		0.086	0.941
PLOC parental control of child’s behavior	-0.139		0.149	0.961
**Panel C. Parenting Practice(N = 564)**				
***Parent-child interactions***				
Reading books	0.176		0.065	0.010
Telling stories	0.196		0.061	0.010
Singing songs	0.086		0.060	0.337
Taking child outside the home for play	-0.055		0.054	0.980
Playing with the child with toys	0.071		0.047	0.644
Naming things, counting, drawing	0.207		0.048	0.010
***Disciplining practices***				
Taking away children’s things	-0.094		0.084	0.980
Limiting time	-0.172		0.117	0.060
Explanation	-0.073		0.090	0.931

**Notes:** In all regressions, we controlled for baseline parental knowledge, attitude and practices, child characteristics, and family characteristics. The scores for parental knowledge and attitudes were internally standardized. All standard errors were clustered at the village level. We also adjusted the multiple hypotheses testing problem using the step-down procedure of Romano and Wolf (2005) to control for the family wise error rate (FWER). The significance levels are as follows: *p < 0.1, **p < 0.05, and ***p < 0.01

## ITT results

Table 3 shows the ITT effects of the interventions on parenting knowledge, attitudes, and practices. The results show that the intervention had no significant impacts on caregiver’s parenting knowledge and attitudes but significantly changed the parenting practices, especially caregiver-child interactions. Compared to caregivers in the control group, caregivers in the treatment group were more likely to read books and tell stories to their children; they were also more likely to name things/count/draw with their children. However, the intervention had little effect on parenting discipline behaviors.

Table 4 shows the effects of receiving monthly feedback on top of the parenting-training treatment. Caregivers in the feedback group had no significant changes in their parenting knowledge and attitudes compared to the no-feedback group, but they were more likely to engage in caregiver-child interactions, such as reading, storytelling, playing with toys, and teaching children to name, count, and draw. For the no-feedback group, the ECD intervention significantly increased the possibility that caregivers would tell stories to children, teach children to name, count, and draw. Overall, monthly feedback obviously increased the intensity of caregiver-child interactions (except for telling stories to children). In addition, regardless of whether feedback was received, the intervention had no effect on discipline behaviors.

## TOT results

The TOT effects presented in Table 5 are consistent with the ITT effects. That is, the intervention had no effect on parenting knowledge and attitudes but significantly increased the caregiver-child interactions, such as reading, stories-telling, and teaching children to name, count and draw. However, unlike the results of the ITT analysis, the frequency of disciplined behaviors, such as stopping what the baby is doing for a period, increased significantly.

## Heterogeneity analysis results

Table 6 shows the heterogeneous ITT effects among different groups. First, the intervention had no effect on parenting knowledge and attitudes regardless of whether the caregiver was internal or external, mother had a higher (above junior high school) or lower (junior high school or below) education level, or delivery model was home-based or center-based. However, when the primary caregiver was not a mother, the intervention increased the feeling of the child’s control over the caregiver’s life. We suspect that the possible reason for this is that non-mother caregivers are usually grandmothers, and the intervention may inadvertently cause them to feel the pressure of having to take children to the ECD center or having home visits every week.

**Table 6:** ITT analysis of treatment on parenting knowledge, attitudes, and practices by subgroups

	Parental locus of control		Mother education		Caregiver type		Delivery model	
	Internal locus of control	External locus of control	High	Low	Non-mother	Mother	Center-based	Home-based
**Panel A. Parenting Knowledge**								
KIDI total score	0.006 (0.076)	0.065 (0.113)	0.017 (0.093)	0.050 (0.087)	-0.054 (0.129)	0.050 (0.081)	-0.048 (0.129)	-0.258 (0.246)
**Panel B. Parenting Attitude**								
PLOC overall	-0.028 (0.115)	0.068 (0.138)	-0.054 (0.100)	0.105 (0.136)	-0.072 (0.207)	0.031 (0.095)	0.023 (0.150)	0.116 (0.327)
PLOC parental efficacy	0.145 (0.096)	0.075 (0.106)	0.067 (0.090)	0.179 (0.130)	-0.054 (0.179)	0.152 (0.096)	0.119 (0.138)	0.132 (0.326)
PLOC parental responsibility	0.043 (0.116)	-0.022 (0.134)	0.003 (0.111)	0.032 (0.147)	-0.151 (0.190)	0.054 (0.096)	0.160 (0.124)	0.113 (0.258)
PLOC child control of parent’s life	0.020 (0.094)	-0.02 (0.129)	-0.005 (0.095)	0.016 (0.087)	0.358* (0.188)	-0.078 (0.067)	-0.083 (0.167)	-0.205 (0.318)
PLOC parental control of child’s behavior	-0.240 (0.150)	0.112 (0.126)	-0.154 (0.118)	0.004 (0.135)	-0.198 (0.156)	-0.063 (0.108)	-0.173 (0.143)	0.179 (0.337)
**Panel C. Parenting Practice**								
***Parent-child interactions***								
Reading books	0.153** (0.060)	0.092 (0.080)	0.148** (0.061)	0.095 (0.066)	0.292** (0.088)	0.087* (0.047)	0.176** (0.077)	0.435** (0.162)
Telling stories	0.193** (0.051)	0.061 (0.073)	0.141** (0.060)	0.129** (0.058)	0.089 (0.082)	0.147*** (0.055)	0.120** (0.060)	0.138 (0.125)
Singing songs	0.130*** (0.048)	-0.025 (0.064)	0.105** (0.049)	0.005 (0.062)	0.111 (0.085)	0.053 (0.043)	0.086 (0.065)	-0.046 (0.145)
Taking child outside home for play	0.036 (0.040)	-0.135** (0.054)	-0.070* (0.039)	0.010 (0.057)	-0.093 (0.090)	-0.024 (0.038)	-0.095** (0.041)	-0.019 (0.176)
Playing with the child with toys	0.046 (0.054)	0.053 (0.050)	0.048 (0.057)	0.050 (0.051)	0.142 (0.095)	0.027 (0.036)	-0.010 (0.073)	-0.197 (0.161)
Naming things, counting, drawing	0.199*** (0.041)	0.073 (0.064)	0.162*** (0.051)	0.123** (0.060)	0.227*** (0.085)	0.127*** (0.042)	0.120 (0.072)	0.183 (0.137)
***Disciplining practices***								
Taking away children’s things	-0.056 (0.085)	-0.065 (0.108)	-0.041 (0.082)	-0.087 (0.079)	-0.191 (0.127)	-0.029 (0.075)	-0.106 (0.117)	0.068 (0.246)
Limiting time	-0.076 (0.106)	-0.135 (0.109)	-0.05 (0.093)	-0.173 (0.153)	-0.303 (0.196)	-0.053 (0.085)	-0.046 (0.130)	-0.463 (0.283)
Explanation	-0.013 (0.075)	-0.104 (0.109)	0.035 (0.082)	-0.172 (0.113)	-0.382** (0.159)	0.027 (0.063)	-0.050 (0.086)	0.191 (0.199)

**Notes:** In all regressions, we controlled for baseline parental knowledge, attitude and practices, child characteristics, and family characteristics. The scores for parental knowledge and attitudes were internally standardized. All standard errors were clustered at the village level. We also adjusted the multiple hypotheses testing problem using the step-down procedure of Romano and Wolf (2005) to control for the family wise error rate (FWER). The significance levels are as follows: *p < 0.1, **p < 0.05, and ***p < 0.01

Second, the ECD intervention had positive impacts on many parenting practices (mainly reading, telling stories, singing songs, teaching children to name/count/draw) of the internal orientation caregivers but not on those of the external orientation caregivers. In addition, the intervention decreased the frequency of taking children to play outside among external oriented caregivers. This indicates that while the intervention increases the frequency of taking children to ECD centers, it may crowd out the frequency of taking children to play outside (crowding out effects) among external oriented caregivers.

Third, mothers with an educational level above junior high school benefited significantly more from the treatment than their less educated counterparts. Interestingly, the intervention also crowded out the frequency of taking children to play outside.

Fourth, when we examined the types of caregivers, the intervention improved the caregiver-child interactions of non-mother caregivers, such as the frequency of reading books and teaching children to name, count, or draw. These effects are weaker in magnitude but wider in dimension when mothers are primary caregivers. As for positive disciplinary behaviors, such as explaining to the child why the behavior is unacceptable, we only found positive effects on non-mother caregivers.

Lastly, the ITT effects on parenting knowledge and attitudes were insignificant regardless of the delivery model of intervention, but the ITT effects on parenting practices were heterogeneous between the two intervention models. Specifically, the intervention increased the frequency of reading books with children in both intervention models, with larger effects among caregivers in the home-based model. Furthermore, the intervention increased the frequency of telling stories to children among caregivers of the center-based model but again crowded out the frequency of taking children to play outside.

Table 7 shows the heterogeneous TOT effects among different groups. First, there was no significant effect on parenting knowledge regardless of caregiver characteristics; however, there was a negative effect on parenting knowledge in the home-based intervention model. One possible reason is that the caregivers of the home-based intervention are likely to be elderly grandparents or less-educated parents. Furthermore, the intervention sites are relatively remote and isolated, so these caregivers have limited access to parenting knowledge and fewer mutual communication opportunities compared to caregivers of the center-based model. When parenting trainers provide guidance on how to play with children, this may challenge their knowledge about parenting. As a result, these caregivers may provide more uncertain answers when asked about parenting knowledge during the research interview, which in turn affects the calculation of parenting knowledge scores. Since the score of parenting knowledge is calculated by dividing the number of questions answered correctly by the total number of questions, an increased number of uncertain answers will decrease the score of parenting knowledge.

**Table 7:** TOT analysis of treatment on parenting knowledge, attitudes, and practices by subgroups

	Parental locus of control		Mother education		Caregiver type		Delivery model	
	Internal locus of control	External locus of control	High	low	Non-mother	mother	center-based	home-based
**Panel A. Parenting Knowledge**								
KIDI total score	-0.040 (0.107)	0.064 (0.172)	0.047 (0.123)	0.064 (0.126)	-0.326 (0.201)	0.071 (0.117)	-0.077 (0.194)	-0.491*** (0.242)
**Panel B. Parenting Attitude**								
PLOC overall	-0.014 (0.151)	0.211 (0.185)	0.168 (0.215)	-0.104 (0.127)	0.275 (0.249)	0.014 (0.133)	0.024 (0.212)	-0.280 (0.508)
PLOC parental efficacy	0.229* (0.136)	0.167 (0.143)	0.243 (0.210)	0.075 (0.118)	0.292 (0.237)	0.178 (0.129)	0.164 (0.196)	-0.299 (0.470)
PLOC parental responsibility	0.074 (0.168)	0.046 (0.187)	0.045 (0.206)	-0.023 (0.150)	-0.284 (0.208)	0.052 (0.138)	0.219 (0.183)	-0.050 (0.382)
PLOC child control of parent’s life	0.074 (0.111)	0.044 (0.180)	0.076 (0.147)	-0.005 (0.124)	0.789** (0.235)	-0.106 (0.099)	-0.062 (0.252)	-0.249 (0.325)
PLOC parental control of child’s behavior	-0.373* (0.214)	0.197 (0.191)	-0.013 (0.210)	-0.224 (0.168)	-0.024 (0.225)	-0.093 (0.164)	-0.294 (0.214)	-0.038 (0.503)
**Panel C. Parenting Practice**								
***Parent-child interactions***								
Reading books	0.203** (0.070)	0.103 (0.113)	0.146 (0.096)	0.205** (0.082)	0.390*** (0.112)	0.100 (0.064)	0.226** (0.105)	0.556*** (0.201)
Telling stories	0.291*** (0.069)	0.034 (0.103)	0.229** (0.077)	0.190** (0.081)	0.087 (0.091)	0.192*** (0.071)	0.141* (0.085)	0.288* (0.172)
Singing songs	0.165** (0.069)	-0.048 (0.095)	-0.008 (0.087)	0.153** (0.066)	0.061 (0.116)	0.057 (0.066)	0.115 (0.096)	-0.231 (0.248)
Taking child outside home for play	0.039 (0.057)	-0.230*** (0.087)	0.013 (0.093)	-0.084 (0.053)	-0.196 (0.130)	-0.038 (0.057)	-0.159*** (0.057)	-0.374 (0.311)
Playing with the child with toys	0.078 (0.074)	0.046 (0.070)	0.073 (0.073)	0.080 (0.082)	0.222 (0.139)	0.035 (0.050)	-0.060 (0.098)	-0.301 (0.209)
Naming things, counting, drawing	0.293*** (0.059)	0.096 (0.089)	0.171** (0.077)	0.228*** (0.070)	0.303*** (0.110)	0.163*** (0.059)	0.147 (0.098)	0.172 (0.208)
***Disciplining practices***								
Taking away children’s things	-0.05 (0.113)	-0.146 (0.138)	-0.138 (0.132)	-0.063 (0.114)	-0.356* (0.183)	-0.039 (0.101)	-0.187 (0.163)	-0.153 (0.274)
Limiting time	-0.114 (0.141)	-0.214 (0.160)	-0.187 (0.213)	-0.145 (0.117)	-0.241 (0.264)	-0.110 (0.117)	-0.143 (0.165)	-0.679 (0.419)
Explanation	-0.031 (0.100)	-0.159 (0.151)	-0.238 (0.162)	0.038 (0.128)	-0.238 (0.186)	0.014 (0.090)	-0.080 (0.113)	0.309 (0.236)

**Notes:** In all regressions, we controlled for baseline parental knowledge, attitude and practices, child characteristics, and family characteristics. The scores for parental knowledge and attitudes were internally standardized. All standard errors were clustered at the village level. We also adjusted the multiple hypotheses testing problem using the step-down procedure of Romano and Wolf (2005) to control for the family wise error rate (FWER). The significance levels are as follows: *p < 0.1, **p < 0.05, and ***p < 0.01

In terms of parenting attitudes, the intervention significantly improved parenting efficacy and reduced the feeling of inability to control their children’s behaviors among internally oriented caregivers. In addition, consistent with the ITT effects, when the primary caregiver was not a mother, the intervention improved the PLOC score of children’s control of their parents’ lives.

In terms of parenting practices, the intervention had a significant effect on caregiver-child interactions (mainly reading, telling stories, singing, teaching children to name, count, or draw) of the internally oriented caregivers but significantly reduced the frequency of children’s outdoor play activities among externally oriented caregivers. There were also different effects on parenting practices among mothers of different education levels, with stronger effects on mothers with lower education levels. These results are inconsistent with the ITT analysis. This is probably because the TOT effect captures the effect of the intervention on the compliers who would only participate because of the provision of the program. These compliers are more likely to be caregivers with lower educational levels. This finding is consistent with the literature that ECD programs are more effective for disadvantaged families in LMICs.

In addition, the intervention improved various caregiver-child interaction activities for mother or non-mother caregivers. For different intervention models, the intervention significantly improved parenting practices (mainly reading books and telling stories), with a greater effect on the home-based intervention model. In addition, the center-based intervention significantly reduced the frequency of outdoor play with children, which is consistent with the crowding-out effects of the ITT analysis.

Regarding discipline styles, the intervention significantly decreased the frequency of disciplining a child by taking away his/her toys or other desirable objects among non-mother caregivers, indicating an improvement in discipline styles for these caregivers.

## DID results

Table 8 shows the changes in the parenting knowledge, attitudes, and practices of caregivers in the treatment and control groups during the baseline and follow-up surveys. Consistent with the ITT results, the DID results showed no significant effects on parenting knowledge and attitudes. Despite this, caregivers’ parenting practices changed significantly. Specifically, parent-child interactions, such as reading, singing, telling stories, playing with the child with toys, naming objects, counting, or drawing with children, increased significantly. Finally, the intervention had no significant effects on caregivers’ discipline behaviors.

**Table 8:** The mean of parenting knowledge, attitudes, and practices between the baseline and follow-up

	Baseline (1)				Follow-up (2)							(2)-(1)		DID		P	
	Treatment		Control		Treatment				Control			Treatment	Control				
***Knowledge***																	
KIDI total score	0.506		0.523		0.477				0.485			-0.030	-0.038	0.008		0.412	
***Attitude***																	
PLOC overall	65.480		65.003		64.225				63.943			-1.255	-1.061	-0.194		0.735	
PLOC parental efficacy	15.808		15.391		14.503				14.003			-1.305	-1.388	0.083		0.732	
PLOC parental responsibility	16.364		16.118		16.007				16.135			-0.358	0.017	-0.374		0.280	
PLOC child control of parent’s life	15.136		15.236		15.526				15.471			0.391	0.236	0.155		0.532	
PLOC parental control of child’s behavior	18.172		18.259		18.189				18.333			0.017	0.074	-0.058		0.845	
***Practice***																	
***Parent-child interactions***																	
Reading books	0.417		0.403		0.692				0.549			0.275	0.146	0.129		0.008	
Telling stories	0.321		0.346		0.613				0.481			0.291	0.136	0.156		0.001	
Singing songs	0.570		0.651		0.639				0.617			0.070	-0.034	0.103		0.022	
Taking child outside the home for play	0.798		0.841		0.742				0.793			-0.056	-0.047	-0.009		0.831	
Playing with the child with toys	0.695		0.793		0.728				0.678			0.033	-0.115	0.148		0.001	
Naming things, counting, drawing	0.444		0.441		0.579				0.454			0.136	0.014	0.122		0.013	
***Disciplining practices***																	
Taking away children’s things	2.685		2.654		2.434				2.492			-0.252	-0.163	-0.089		0.345	
Limiting time	2.679		2.600		2.076				2.136			-0.603	-0.464	-0.138		0.175	
Explanation	1.689		1.712		1.450				1.498			-0.238	-0.214	-0.025		0.779	

## Conclusion

This study used a cluster-randomized controlled trial to evaluate the effects of a government-led ECD program on the parenting knowledge, attitudes, and practices of caregivers in a poverty-stricken area in Northwest China. This study found that while the program had no substantial effects on the parenting knowledge and attitudes of caregivers, it significantly improved caregivers’ parenting practices, with heterogeneous effects among different groups. On average, the ITT effects show that internal orientation caregivers, mothers with higher education levels, and mothers being the primary caregivers were more likely to benefit from the intervention. In contrast, the TOT effects show that, among compliers to the program, the intervention also had a significant effect on caregiver-child interactions among mothers with lower education levels. Lastly, the parenting training program had greater effects on parenting practices among caregivers of the home-based delivery model. These results suggest that, at least in the short run, the program can directly change caregivers’ parenting practices without changing their knowledge and attitudes. Future studies are needed to investigate whether parenting knowledge and attitudes can change over the long run, how the caregiver practices moderate child outcomes, and also the extent to which practices change among high-risk parents relates to child outcomes. The study of treatment effects on ECD outcomes is also needed in future studies and is under analysis by the research team.

We want to emphasize that, although the ITT effect on parenting practices (the average treatment effect) were stronger for mothers with higher education, the TOT effect on parenting practices (the local average treatment effect, LATE) were stronger for mother with less education. That is, even though on average the program helped mothers with higher education, but among complier families (i.e., families that will only participate if the program is provided and will not participate otherwise), the program benefited mothers with less education.

We also want to elaborate on the findings that the program did not significantly change parenting knowledge and attitudes, but significantly changed parenting practices. If we look at the literature, many studies have found that the parenting knowledge of caregivers is closely linked to ECD. According to the hypothesis on the transmission mechanism of knowledge to practices, ECD-related parenting knowledge will affect caregivers’ behaviors and interactions with the child (Huang et al. 2005; Fry 2011; Al-Hassan and Landsford, 2011), thereby affecting the child’s development. Furthermore, according to the *Knowledge-Attitude-Practice (KAP) Theory* (Schwartz 1976), there is a progressive relationship between knowledge, belief (attitude), and behavior: the caregiver can only begin to think proactively after gaining relevant parenting knowledge. Consequently, driven by a strong sense of responsibility, parenting attitudes may change, leading to a change in practice. We want to point out that, although there is no explicit knowledge intervention component in the design of the experiment, based on the above *KAP Theory* and findings, we believe that we should explore the effect on parenting knowledge because our parenting training courses and the promotion of the importance of ECD during the interaction between parenting trainers and caregivers may increase awareness of the importance of ECD among caregivers. In turn, this may encourage caregivers to focus on and acquire ECD-related knowledge from other sources.

The findings of this study apparently did not follow the above-mentioned path of behavioral change. The findings indicate that, at least in the short run, a program can directly change caregivers’ parenting practices without changing their knowledge and attitudes. Several reasons may explain these findings. First, the intervention content of this study focused specifically on caregivers’ parenting practices, and there was no systematic training on the parenting knowledge of caregivers. Even though the staff could have spread some basic parenting knowledge in their daily interactions with families during the intervention, they would only have concentrated on raising caregivers’ understanding of the importance of ECD and the value of the parent-child interaction. This may be one of the key reasons why the intervention did not significantly improve the parenting knowledge of the primary caregivers but significantly improved parenting practices. Second, the intervention had no effect on the caregivers’ parenting attitudes. This may be because changing individual perceptions is a long-term process that cannot be influenced by short-term intervention. Furthermore, as previously noted, this study did not intervene in the caregivers’ parenting knowledge, so it can be expected that the caregivers’ parenting attitudes would not change significantly.

This study had a few limitations. First, we want to point out that our findings are from a short-term study of the intervention, and follow-up studies are needed to verify the long-term effects of the program. In the long run, parenting knowledge and attitudes might change and create lasting effects on parenting practices. Second, this study was conducted in a specific county in China, which may raise concerns about the external validity of our findings. Third, multiple measures of parenting knowledge and attitudes should be employed in future studies to ensure the robustness of the results. Last, measures of caregiver changes were caregiver-reported, which may be subject to measurement errors. We tried very hard to avoid such error in key variables during the survey by using standardized survey questionnaire and procedures, and the questionnaires we used on parental outcome measures had high reliability and validity. We believe that the measurement error may still exist, but its level should be in the acceptable range.

It should be noted that the ultimate goal of the intervention program studied in this paper is to identify two effects: the program effect on ECD and its mechanism. This paper mainly focuses on the mechanism of the program, that is, the effect of intervention on parenting knowledge, attitudes, and practices. We think this is key to understand the mechanism by which the intervention affect children’s development (Tang et al., 2020). We understand that outcomes on ECD are important to understand the importance of parental practices and so on, we will report on the program impact on ECD in full detail in another article.

The early development of children under the age of three is of great significance to the development of individuals, families, and society. Previous studies have pointed out that, especially in LMICs, the benefits of high-quality ECD as part of social security policies led by the government are substantial and high (Schweinhart and Weikart 1981; Schweinhart et al. 1985). This study provides new evidence that the government-led, multiple-delivery-model, and full-coverage ECD program can assist low-income families in raising their awareness of the value of ECD, learning parenting practices that promote ECD, and making better decisions and choices in early human capital investment.

## Electronic supplementary material

Below is the link to the electronic supplementary material.

Supplementary Material 1

## Data Availability

The dataset(s) supporting the conclusions of this study are not publicly available due to confidentiality. However, this information is available from the corresponding author upon request.

## Declarations

### Trial registration

ISRCTN registry – Registration date: 30/05/2018 - Registration number: ISRCTN16736104.
