# Supplementary material for: The effect of ECD program on the caregiver’s parenting knowledge, attitudes, and practices: based on a cluster-randomized controlled trial in economically vulnerable areas of China
Source: BMC Public Health. 2022 Oct 24;22:1958. doi: 10.1186/s12889-022-14268-5 (PMC9590161; doi:10.1186/s12889-022-14268-5)
Supplement: Supplementary file 1 — Supplementary Material 1 [file 12889_2022_14268_MOESM1_ESM.docx]

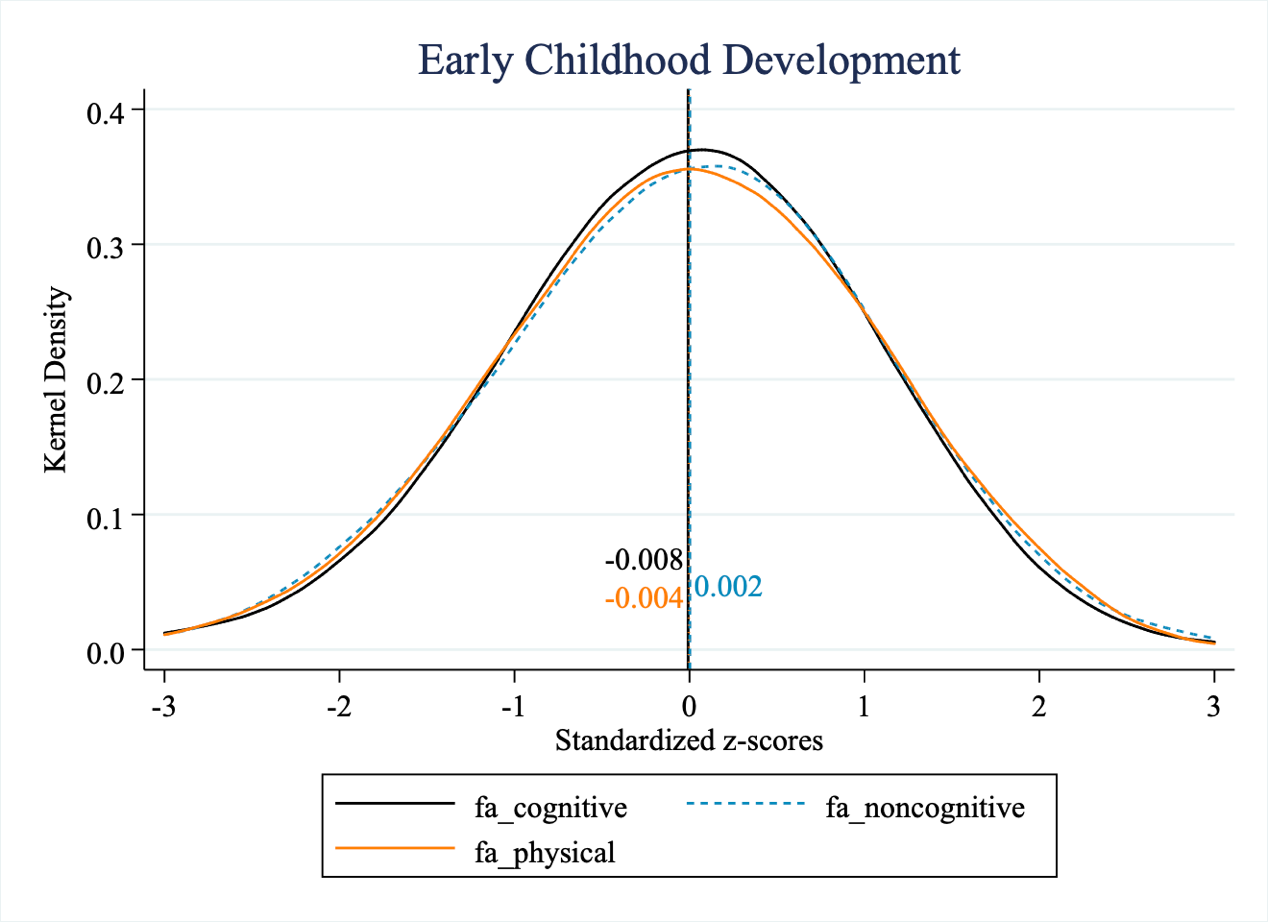

**Appendix Figure 1.** **Distribution of Standardized ECD Factor Scores**

Notes: This figure plots the factor 1 z-scores distribution of cognitive, non-cognitive, and physical development. According to the distribution of standardized ECD factor scores, it can be concluded that the baseline means of cognitive and physical development of infants and toddlers in the sampled area is slightly lower than the mean of the reference population, and the mean score of non-cognitive development is slightly higher than the reference population. According to the distribution of follow-up standardized ECD factor scores, the distribution shows a significant rightward shift in cognitive scores, and a slight rightward shift in non-cognitive and physical scores, compared to the distribution at baseline. This indicates that the early childhood development outcomes did improve after intervention.

**Appendix Table 1. The summary statistics of the CREDI, ASQ-3, and ASQ:SE**

| Measures | mean | sd | min | max |
| --- | --- | --- | --- | --- |
| CREDI(N=963) |  |  |  |  |
| cognitive_raw | 19.536 | 7.205 | 2.000 | 31.000 |
| language_raw | 20.737 | 11.513 | 0.000 | 40.000 |
| motor_raw | 30.355 | 7.059 | 3.000 | 40.000 |
| social-emotional_raw | 13.549 | 5.367 | 2.000 | 22.000 |
| motor_z | -0.019 | 1.027 | -4.414 | 6.069 |
| cognitive_z | 0.044 | 1.106 | -3.987 | 5.887 |
| language_z | 0.330 | 1.077 | -3.503 | 6.951 |
| social-emotional_z | 0.277 | 1.068 | -4.003 | 5.101 |
| overall_z | 0.158 | 0.953 | -4.028 | 6.114 |
| motor_delay_sd | 0.159 | 0.366 | 0.000 | 1.000 |
| cogitive_delay_sd | 0.163 | 0.370 | 0.000 | 1.000 |
| language_delay_sd | 0.106 | 0.308 | 0.000 | 1.000 |
| social-emotional_delay_sd | 0.096 | 0.294 | 0.000 | 1.000 |
| overall_delay_sd | 0.103 | 0.304 | 0.000 | 1.000 |
| ASQ-3(N=947) |  |  |  |  |
| problem solving_raw | 46.426 | 12.729 | 0.000 | 60.000 |
| communication_raw | 45.517 | 13.659 | 0.000 | 60.000 |
| gross motor_raw | 47.687 | 13.953 | 0.000 | 60.000 |
| fine motor_raw | 41.616 | 14.666 | 0.000 | 60.000 |
| personal-social_raw | 44.951 | 11.678 | 10.000 | 60.000 |
| problem solving_st | 0.018 | 0.992 | -3.321 | 1.354 |
| communication_st | 0.017 | 0.992 | -3.879 | 1.601 |
| gross motor_st | 0.005 | 0.987 | -4.571 | 1.783 |
| fine motor_st | 0.014 | 0.992 | -2.866 | 1.461 |
| personal-social_st | 0.015 | 0.996 | -3.547 | 1.480 |
| problem solving_delay_sd | 0.224 | 0.417 | 0.000 | 1.000 |
| communication_delay_sd | 0.223 | 0.416 | 0.000 | 1.000 |
| fine motor_delay_sd | 0.393 | 0.489 | 0.000 | 1.000 |
| gross motor_delay_sd | 0.283 | 0.451 | 0.000 | 1.000 |
| personal-social_delay_sd | 0.302 | 0.459 | 0.000 | 1.000 |
| problem solving_delay_2sd | 0.110 | 0.313 | 0.000 | 1.000 |
| communication_delay_2sd | 0.075 | 0.263 | 0.000 | 1.000 |
| fine motor_delay_2sd | 0.187 | 0.390 | 0.000 | 1.000 |
| gross motor_delay_2sd | 0.141 | 0.349 | 0.000 | 1.000 |
| personal-social_delay_2sd | 0.108 | 0.310 | 0.000 | 1.000 |
| ASE:SE(N=964) |  |  |  |  |
| total_raw | 62.265 | 44.539 | 0.000 | 255.000 |
| asqse_delay | 0.498 | 0.500 | 0.000 | 1.000 |

Notes：(1) From the baseline CREDI results, the mean of the overall development score (standardized score Z=0.158) of the sampled children was higher than the mean of the reference population. The mean scores of cognitive, language, and social-emotional development were also higher than the population mean. Only the mean score of motor development was lower than the mean. Follow the definition of developmental delay (i.e., a development score that is one standard deviation below the mean of the reference population), 16.3% of the sample had a risk of delay in cognitive development, and these ratios of development delays in language, social-emotional, and motor development was10.6%, 9.6%, and 15.9% respectively. (2) From the baseline ASQ-3 results, the mean scores of each domain were all higher than the mean of the reference population. Among them, 22.4% of the sample had a risk of slight delay (<1 SD) and 11% had a risk of serious delay (<2 SDs) in problem-solving. These ratios of slight delay and serious delay were 22.3% and 7.5% for the communication delay; 39.3%, and 18.7% for the fine motor; 28.3% and 14.1% for the gross motor; and 30.2% and 10.8% for the personal-social. (3) From the baseline ASQ:SE results, nearly half of the sampled children were at the risk of social-emotional development delay.

**Appendix Table 2. Summary of baseline parenting knowledge, attitudes, and practices among different caregivers**

|  | Non-mother | Mother | p-value |
| --- | --- | --- | --- |
| ***Knowledge*** |  |  |  |
| KIDI total score | 0.486 | 0.522 | 0.000 |
|  | (0.008) | (0.004) |  |
| ***Attitude*** |  |  |  |
| PLOC overall | 65.955 | 65.183 | 0.081 |
|  | (0.415) | (0.181) |  |
| PLOC parental efficacy | 16.519 | 15.281 | 0.000 |
|  | (0.236) | (0.080) |  |
| PLOC parental responsibility | 15.870 | 16.338 | 0.053 |
|  | (0.187) | (0.132) |  |
| PLOC child control of parent's life | 15.490 | 15.110 | 0.058 |
|  | (0.176) | (0.077) |  |
| PLOC parental control of child's behavior | 18.096 | 18.454 | 0.181 |
|  | (0.253) | (0.090) |  |
| ***Parenting Practices*** |  |  |  |
| ***Parent-child interactions*** |  |  |  |
| Reading books | 0.424 | 0.440 | 0.625 |
|  | (0.045) | (0.025) |  |
| Telling stories | 0.352 | 0.357 | 0.881 |
|  | (0.042) | (0.024) |  |
| Singing songs | 0.514 | 0.646 | 0.006 |
|  | (0.054) | (0.029) |  |
| Taking child outside the home for play | 0.779 | 0.846 | 0.031 |
|  | (0.030) | (0.017) |  |
| Playing with the child with toys | 0.655 | 0.791 | 0.001 |
|  | (0.043) | (0.021) |  |
| Naming things, counting, drawing | 0.419 | 0.448 | 0.478 |
|  | (0.040) | (0.022) |  |
| ***Disciplining practices*** |  |  |  |
| Taking away children's things | 2.759 | 2.687 | 0.164 |
|  | (0.047) | (0.028) |  |
| Limiting time | 2.679 | 2.666 | 0.851 |
|  | (0.087) | (0.053) |  |
| Explanation | 1.686 | 1.691 | 0.926 |
|  | (0.050) | (0.038) |  |
| N | 296 | 668 |  |

**Appendix Table 3. Summary of characteristics of the participants and**

**balance checks for stayers**

|  | Control | Treatment | p-value |
| --- | --- | --- | --- |
| ***Individual characteristics*** |  |  |  |
| Gender | 0.469 | 0.513 | 0.277 |
|  | (0.028) | (0.030) |  |
| Months | 20.566 | 20.041 | 0.537 |
|  | (0.701) | (0.461) |  |
| Premature | 0.042 | 0.048 | 0.752 |
|  | (0.011) | (0.014) |  |
| Minority | 0.080 | 0.006 | 0.050 |
|  | (0.037) | (0.004) |  |
| The child was firstborn | 0.730 | 0.752 | 0.527 |
|  | (0.026) | (0.028) |  |
| Low birth weight (<=2.499 kg) | 0.042 | 0.049 | 0.727 |
|  | (0.012) | (0.014) |  |
| The first factor of cognitive development | 0.028 | -0.050 | 0.515 |
|  | (0.061) | (0.095) |  |
| The first factor of non-cognitive development | 0.129 | -0.207 | 0.027 |
|  | (0.082) | (0.124) |  |
| The first factor of physical development | 0.077 | -0.164 | 0.058 |
|  | (0.071) | (0.100) |  |
| ***Family characteristics*** |  |  |  |
| The primary caregiver is a mother | 0.794 | 0.742 | 0.199 |
|  | (0.029) | (0.030) |  |
| Mother's education level (> 9 years of formal education) | 0.391 | 0.412 | 0.818 |
|  | (0.073) | (0.067) |  |
| Age of the mother (years) | 29.462 | 29.595 | 0.811 |
|  | (0.428) | (0.346) |  |
| Hukou of parents | 1.362 | 1.342 | 0.892 |
|  | (0.110) | (0.120) |  |
| Whether is *Dibao* | 0.102 | 0.144 | 0.238 |
|  | (0.025) | (0.026) |  |
| Distance from the home to township seat | 1.442 | 1.482 | 0.854 |
|  | (0.115) | (0.194) |  |
| N | 311 | 314 |  |

**Appendix Table 4. Summary of parenting knowledge, attitudes, and practices and balance checks for stayers**

|  | Control | Treatment | p-value |
| --- | --- | --- | --- |
| ***Knowledge*** |  |  |  |
| KIDI total score | 0.520 | 0.504 | 0.021 |
|  | (0.005) | (0.005) |  |
| ***Attitude*** |  |  |  |
| PLOC overall | 64.974 | 65.604 | 0.137 |
|  | (0.334) | (0.263) |  |
| PLOC parental efficacy | 15.412 | 15.892 | 0.025 |
|  | (0.173) | (0.118) |  |
| PLOC parental responsibility | 16.101 | 16.376 | 0.347 |
|  | (0.218) | (0.187) |  |
| PLOC child control of parent's life | 15.218 | 15.188 | 0.879 |
|  | (0.128) | (0.138) |  |
| PLOC parental control of child's behavior | 18.244 | 18.166 | 0.716 |
|  | (0.178) | (0.128) |  |
| ***Parenting Practices*** |  |  |  |
| ***Parent-child interactions*** |  |  |  |
| Reading books | 0.392 | 0.412 | 0.736 |
|  | (0.044) | (0.037) |  |
| Telling stories | 0.343 | 0.313 | 0.566 |
|  | (0.035) | (0.039) |  |
| Singing songs | 0.637 | 0.559 | 0.152 |
|  | (0.047) | (0.034) |  |
| Taking child outside the home for play | 0.837 | 0.792 | 0.255 |
|  | (0.022) | (0.032) |  |
| Playing with the child with toys | 0.788 | 0.696 | 0.015 |
|  | (0.027) | (0.029) |  |
| Naming things, counting, drawing | 0.438 | 0.431 | 0.892 |
|  | (0.032) | (0.035) |  |
| ***Disciplining practices*** |  |  |  |
| Taking away children's things | 2.649 | 2.684 | 0.601 |
|  | (0.051) | (0.047) |  |
| Limiting time | 2.613 | 2.677 | 0.512 |
|  | (0.066) | (0.072) |  |
| Explanation | 1.741 | 1.687 | 0.545 |
|  | (0.069) | (0.044) |  |
| N | 311 | 314 |  |

**Appendix Table 5. Intervention activities**

|  | Total  (n=643) | Treatment  (n=324) | Control  (n=319) |
| --- | --- | --- | --- |
| No. of one-to-one sessions |  |  |  |
| Average per child | 5.85(8.39) | 10.59(9.13) | 1.03(3.38) |
| No. of visits to the ECD center | | | |
| Average per child | 13.62(25.65) | 23.93(31.32) | 3.14(10.64) |
| No. of hours spent at the ECD center | | | |
| Average per child | 16.75(37.72) | 29.91(48.53) | 3.39(11.16) |
